# Draft Genome Sequence of *Phenylobacterium immobile* Strain E (DSM 1986), Isolated from Uncontaminated Soil in Ecuador

**DOI:** 10.1128/genomeA.00420-15

**Published:** 2015-05-14

**Authors:** Ondrej Reznicek, Francisca Luesken, Sandra J. Facey, Bernhard Hauer

**Affiliations:** a Institute of Technical Biochemistry, University of Stuttgart, Stuttgart, Germany; b Research Unit Environmental Genomics, Helmholtz Centre Munich, Munich, Germany

## Abstract

We report the draft genome sequence of 3.3 Mb and the sequence (19.2 kb) of a natural plasmid isolated from *Phenylobacterium immobile* strain E (DSM 1986), able to degrade xenobiotic compounds as the sole carbon source. The sequences reveal a large number of novel Rieske nonheme iron aromatic ring-hydroxylating oxygenases (RHOs).

## GENOME ANNOUNCEMENT

Herbicides such as chloridazon or aromatic compounds of medical use, such as the xenobiotics antipyrine and diclofenac, are considered as pollutants when exposed to the environment (1). Microbial degradation of such pollutants often occurs via initial hydroxylation of the phenolic moiety using dioxygenases (DOs) to make it enzymatically accessible for further degradation (2). *Phenylobacterium immobile* (DSM 1986), a soil bacterium whose strains are found on various continents (3) and is capable of degrading the xenobiotic compounds chloridazon and antipyrine as the sole carbon source (4), has been examined for its enzymatic content to explore novel ring-hydroxylating oxygenases (RHOs).

The draft genome of *P. immobile* strain E was obtained using Illumina shotgun and mate-pair sequencing, which resulted in paired-end reads of 250 bp. Raw data, consisting of 9,340,262 reads, were clipped and trimmed with CASAVA Illumina software. Reads with more than one *N* or a final length < 20 bases were removed. Quality-trimmed reads were error corrected using Musket version 1.0.6, with a 21-bp k-mer size for correction. The error-corrected reads were digitally normalized by normalize_by_median.py from the khmer package version 0.3, with a coverage cut off from 80, and reads <21 bases were discarded, yielding a data set of 1,845,682 reads. To assemble the digitally normalized reads into scaffolds, Allpaths LG release 47547 software was used. Gap closure and refinement of the scaffolds was done with SOAP GapClosure version 1.12 and SEQuel version 1.0.2, respectively. All reads were aligned against the assembled scaffolds with Bowtie2 version 2.1.0. Assembly of the digitally normalized reads resulted in 117 scaffolds in total, with a G+C content of 67%. Comprising 2.88 Mb, the largest scaffold_1 was assembled from 1,494,972 reads, which is 86% of the total reads. Scaffold_2 consists of 269 kb, followed by scaffold_3 (103.8 kb), scaffold_4 (43.5 kb), and scaffold_5 (19.2 kb) representing a natural plasmid and scaffold_6 (13 kb). Remaining scaffolds range below 10 kb or contain gaps.

To search for open reading frames (ORFs), the Glimmer program (5) was used. The ORFs were blasted against the NCBI-nonredundant database, Gen3D, SMART, Pfam, TIGRFam, SUPERFAMILY, HAMP, PIRSF, COILS, and InterProScan. Out of 3,520 identified proteins, 18 ORFs coding for α-subunits of RHOs were found, showing only low identity (<36%) to known enzymes. Additionally, only 1 RHO β-subunit ORF and 6 ORFs coding for extradiol-DOs were identified. Being the key enzymes regarding degradation of aromatic compounds, no traditional gene clusters were identified. Many of the α-subunit RHOs and corresponding ferredoxins and reductases (6) were found to be located at different positions, mainly within scaffold_1. Also, one RHO α-subunit ORF was found to be located within the identified plasmid. The complex genomic organization of *P. immobile* with its astonishing number of oxygenases harbors a source of novel hydroxylating enzymes for applications in biotechnology (7) and organic synthesis and will be further studied.

### Nucleotide sequence accession numbers. 

The scaffolds of this whole-genome shotgun project have been deposited in the European Nucleotide Archive (ENA) under the accession numbers CVJQ01000001 to CVJQ01000006 (CVJQ01000001.1 and CVJQ01000006.1, respectively).
